# An Integrated Germanium-Based THz Impulse Radiator with an Optical Waveguide Coupled Photoconductive Switch in Silicon

**DOI:** 10.3390/mi10060367

**Published:** 2019-05-31

**Authors:** Peiyu Chen, Mostafa Hosseini, Aydin Babakhani

**Affiliations:** 1 Department of Electrical and Computer Engineering, Rice University, Houston, TX 77005, USA; 2 Department of Electrical and Computer Engineering, University of California at Los Angeles, Los Angeles, CA 90095, USA; mostafahosseini@ucla.edu (M.H.); aydinbabakhani@ucla.edu (A.B.)

**Keywords:** germanium, integrated optics, optoelectronics, photoconductivity, silicon photonics, terahertz

## Abstract

This paper presents an integrated germanium (Ge)-based THz impulse radiator with an optical waveguide coupled photoconductive switch in a low-cost silicon-on-insulator (SOI) process. This process provides a Ge thin film, which is used as photoconductive material. To generate short THz impulses, N++ implant is added to the Ge thin film to reduce its photo-carrier lifetime to sub-picosecond for faster transient response. A bow-tie antenna is designed and connected to the photoconductive switch for radiation. To improve radiation efficiency, a silicon lens is attached to the substrate-side of the chip. This design features an optical-waveguide-enabled “horizontal” coupling mechanism between the optical excitation signal and the photoconductive switch. The THz emitter prototype works with 1550 nm femtosecond lasers. The radiated THz impulses achieve a full-width at half maximum (FWHM) of 1.14 ps and a bandwidth of 1.5 THz. The average radiated power is 0.337 μW. Compared with conventional THz photoconductive antennas (PCAs), this design exhibits several advantages: First, it uses silicon-based technology, which reduces the fabrication cost; second, the excitation wavelength is 1550 nm, at which various low-cost laser sources operate; and third, in this design, the monolithic excitation mechanism between the excitation laser and the photoconductive switch enables on-chip programmable control of excitation signals for THz beam-steering.

## 1. Introduction

Sandwiched between traditional microwave and optical spectrums, terahertz (THz) technology has attained great scientific interest in recent decades. Compared to a THz continous-wave (CW) signal, THz impulses feature ultra-wide bandwidth, usually larger than 1 THz. This wide frequency band allows THz impulse to be used for various applications, such as, biology and medicine sciences [1], environmental monitoring [2,3], chemical sensing [4,5], high-resolution three-dimensional imaging [6,7,8,9], nondestructive evaluation [10], and high-speed wireless communication link [11].

Researchers have been investigating various technologies that can produce high power and wideband THz impulses. There are two technical solutions. One solution is to use fully-electronics technology; the other is to rely on optoelectronics methods. In recent years, silicon-based fully-electronics THz impulse radiators have been reported using CMOS or BiCMOS process technologies [12,13,14,15,16]. These fully-electronics devices produce picosecond impulses that cover the lower end of THz spectrum (less than 1.1 THz). Additionally, these designs feature the benefits of low cost, high scalability, and low power consumption.

**Figure 1:**
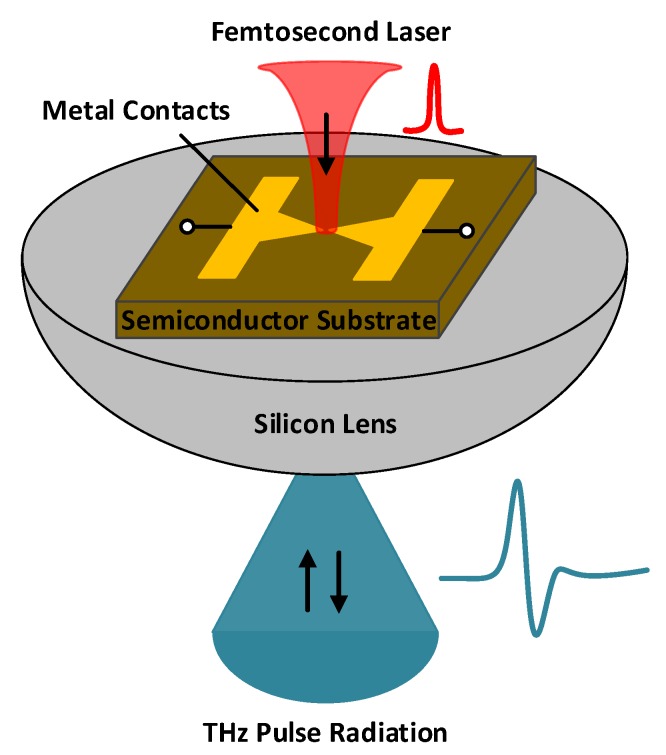
A conventional THz photoconductive antenna (PCA) emitter.

The more widely used and traditional solution of THz impulse generation is photoconductive antennas (PCAs) based on optoelectronics technology [17]. As shown in Figure 1, a conventional THz PCA has two parts of metal contacts fabricated on a photoconductive semiconductor substrate, with a gap between the two metal contacts. The two metal contacts also operate as on-chip THz antennas. Conventional THz PCAs are usually triggered by free-space femtosecond laser, which is incident onto the photoconductive semiconductor substrate through the gap between the metal contacts. The semiconductor substrate has appropriate bandgap energy so that the incident femtosecond laser pulses are absorbed and photocarriers are generated. A DC bias voltage is applied across the gap through the metal contacts, building up electric fields in the substrate below the gap. Consequently, photocarriers drift and are eventually collected by the metal contacts before photocarrier recombinations occur. The induced ultrafast photocurrent drives the on-chip THz antennas that produce THz impulse radiations. A silicon lens is usually attached to the backside of the substrate to increase radiation efficiency.

Admittedly, researchers have attempted to exploit the unique properties of various nanostructures, such as plasmonic structures [18,19] and optical nano-antennas [20,21], to improve radiation bandwidth, radiated power, and efficiency [22,23], but they all share the same fundamental limitations on the photoconductive semiconductor substrate and free-space optical excitation scheme:

First, to ensure large radiation bandwidth, the semiconductor substrate must be an ultrafast photoconductive material that should exhibit short photocarrier lifetime and high carrier mobility. Radiation-damaged silicon-on-sapphire (RDSOS) and low-temperature-grown GaAs (LT-GaAs) are widely used for THz PCAs [24]. Due to the complicated fabrication procedures of these materials, the cost of conventional THz PCAs is high. Another limitation on substrate material is that these materials are usually not compatible with 1550 nm laser excitations. There are various low-cost laser sources at 1550 nm regime for fiber-optic communications. Therefore, designing a THz PCA that operates with a 1550 nm laser source is a cost-effective strategy to reduce the cost further. Due to the aforementioned limitations, the first technical challenge of this work is to use low-cost and ultrafast photoconductive semiconductor materials that operate at 1550 nm region.

Conventional PCAs require free-space optical excitation scheme, which also exhibits two limitations. First, this excitation scheme requires accurate optical alignments to ensure that the femtosecond laser is aligned onto the tiny gap between the metal contacts. Optical alignment has stringent requirements on system stability, and consequently, is not suitable for portable applications. Second, because the optical excitation signal propagates in the free space before it is absorbed by the substrate, programmable control on the excitation signal is usually performed in the free space by using a mechanical translation stage with retroreflector mirrors [2,10]. Therefore, the free-space optical excitation scheme in conventional THz PCAs prevents the implementation of fully integrated THz PCA phased arrays, resulting in the second technical challenge of this work: design an on-chip optical excitation scheme for THz impulse radiator chips.

In this work, we present a Germanium (Ge)-based THz impulse radiator in silicon that resolves the aforementioned limitations. Following the operation principle of THz photoconductive antennas [17], our proposed design features three advantages: first, due to the bandgap energy of Ge, which is used as the photoconductive substrate, the prototype THz impulse radiator can be excited by 1550 nm femtosecond laser sources. As a result, various low-cost laser sources in this wavelength regime can be used to reduce cost. Second, it incorporates a waveguide-coupled photoconductive switch that provides a monolithic interaction between the optical excitation signal and the photoconductive substrate. This novel coupling mechanism eliminates the existing obstacle of achieving on-chip programmable control of excitation laser. Third, this optoelectronics device was batch fabricated by a silicon photonics process foundry, similar to microelectronic chip tape-out: we completed device design using the computer-aided design (CAD) tool and sent the design layout file to the foundry for batch fabrication. In this work, there is no need for post-processing in clean room, which can significantly reduce the cost for mass production.

This paper is an extension of [25] with extensive details of device design and analysis of the simulated and measured results. The remaining context of this paper is organized as follows. Section 2 presents the design techniques and system architecture of the prototype THz impulse emitter. Section 3 describes the measurement results, followed by conclusions in Section 4.

## 2. Design Techniques and System Architecture

The silicon photonics process technology used in this work implements photonics devices using CMOS-compatible technologies. As a result, this technology can significantly reduce the cost of integrated photonics devices in mass production. The silicon photonics process technology is provided by the Institute of Microelectronics (IME), Agency for Science Technology and Research (A*STAR), Singapore [26]. This process has a silicon-on-insulator substrate, and it provides a module library with various pre-designed integrated passive devices, such as single-mode optical waveguides and optical grating couplers. Apart from the passive devices, Ge-based active devices, such as ring modulators and photodetectors, are available for use in this process. Both passive and active devices are optimized for the 1550 nm regime.

### 2.1. Ultrafast Germanium Thin Film

The silicon photonics process technology used in the work provides Ge as the photoconductive material at 1550 nm wavelength. As discussed in Section 1, there are two requirements for the photoconductive materials of THz impulse radiators. The first requirement is that the photocarrier lifetime of the material should be on the order of sub-picoseconds; the second requirement is that the photocarrier mobility should be high enough to produce large photocurrent. A previous work [27] has demonstrated that photocarrier lifetime of Ge thin films can be reduced to the order of sub-picoseconds by implanting O${}^{+}$ ions without sacrificing photocarrier mobility, which can remain at about 100 cm${}^{2}$/(Vs). These results can be applied to the present investigation. The process technology can fabricate Ge thin films with 500 nm thickness, and it provides phosphorus implant with a dose of 4 × 10${}^{15}$ cm${}^{-2}$. Therefore, the first technical challenge of this work, low-cost and ultrafast photoconductive semiconductor material that operates with 1550 nm excitation laser source, is resolved.

### 2.2. Waveguide-Coupling THz Photoconductive Switch

**Figure 2:**
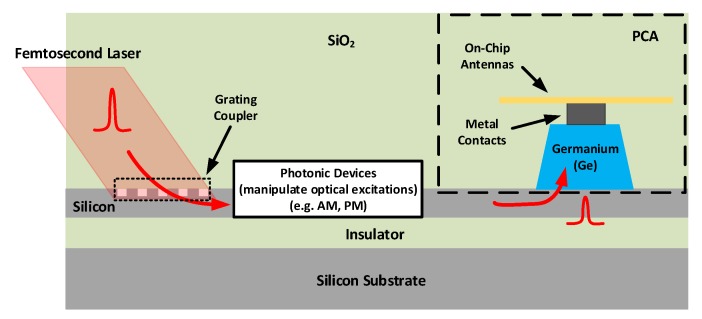
A conceptual illustration of the proposed optical waveguide coupling excitation scheme.

The second technical challenge is to design an on-chip optical excitation scheme for THz impulse radiators. Figure 2 demonstrates a conceptual illustration of the proposed waveguide-coupling excitation mechanism. In this scheme, the free-space 1550 nm femtosecond laser is firstly coupled into the on-chip optical waveguides through an integrated optical grating coupler. Then, the femtosecond laser propagates in the optical waveguide and reaches at silicon photonics devices that can modulate the excitation optical signal by performing amplitude modulation or phase modulation. The modulated femtosecond laser continues to propagate in the optical waveguide until it is absorbed by the Ge thin film, which excites the photoconductive switch that drives the on-chip THz antennas to radiate THz impulses.

**Figure 3:**
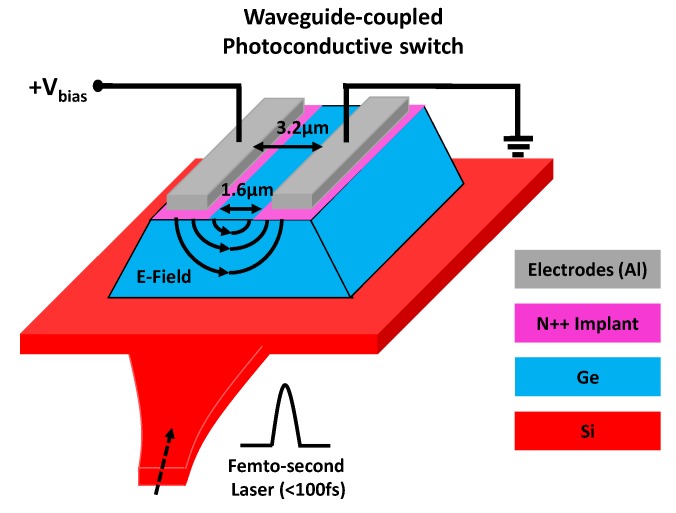
Structure of the proposed waveguide-coupling THz photoconductive switch.

Figure 3 presents the structure of the implemented Ge-based waveguide-coupling photoconductive switch. The femtosecond excitation laser travels to the photoconductive switch through an integrated optical waveguide. A tapered transition is designed between the optical waveguide and the switch to reduce undesired optical reflections. A Phosphorus-doped Ge thin film is grown on the silicon layer. The N++ (Phosphorus) implant layer is split into two parts to prevent a large dc current produced under dc biasing. To increase the transient response speed of the photoconductive switch, the spacing between the two metal electrodes is set to the minimum value allowed by the DRC rule of the process technology. Compared with conventional THz photoconductive switches, the proposed waveguide-coupling solution exhibits a significant advantage: In this design, the femtosecond laser arrives at the photoconductive switch from the substrate side rather than through the gap between the metal electrodes as in conventional designs. Therefore, when a small spacing between the metal electrodes is required for enhancing the transient response speed, the optical excitation signal will not be blocked by the small gap, and consequently, complicated plasmonics-related simulation and design can be avoided. As shown in Figure 3, on-chip THz antennas are connected to the two metal electrodes.

**Figure 4:**
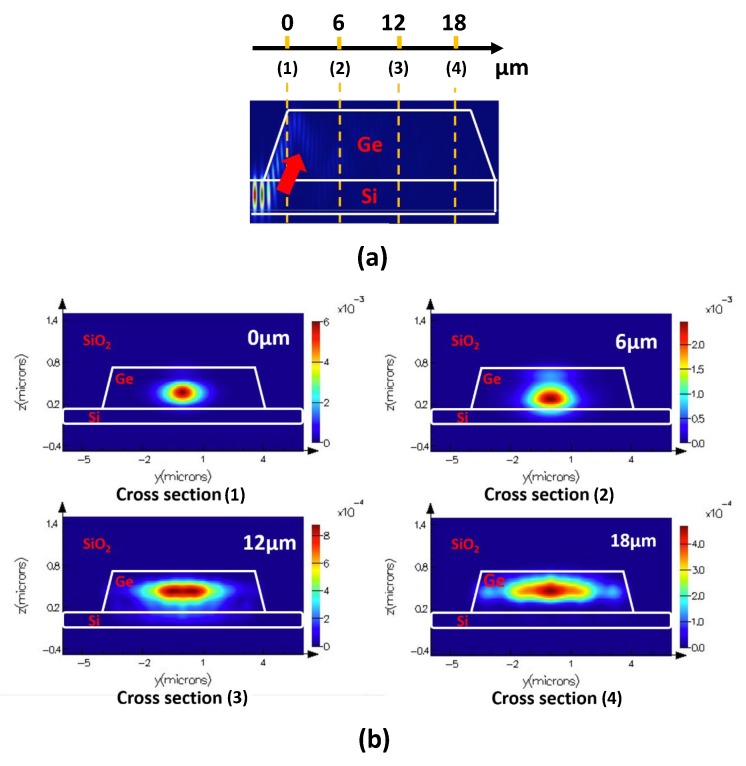
(**a**) Simulated optical absorption at 1550 nm within the proposed photoconductive switch. (**b**) Simulated 1550 nm optical mode distributions at different propagation distances within the proposed photoconductive switch.

The Ge thin film is optimized to increase both absorption efficiency and conversion efficiency at 1550 nm regime. The length of Ge thin film is designed to be 20 μm to ensure high absorption efficiency. Figure 4a shows that the 20 μm Ge thin film can absorb almost all the incident optical excitation signal at 1550 nm. The conversion efficency boost can be explained by investigating the optical mode distributions at different propagation distances within the photoconductive switch at 1550 nm. As shown in Figure 4b, the optical mode size is expanded transversly along propagation in the Ge thin film. As a result, more photocarriers are generated at closer locations to the metal electrodes, followed by being converted to photocurrent before recombination occurs. As a result, the conversion efficiency from photocarriers to photocurrent in the Ge thin film is increased.

The efficiency of THz photoconductive switches can be potentially enhanced by introducing artificial 3D architectures that exhibit tailored optoelectronic properties. Artificial 3D structures have been proposed and demonstrated for various THz applications, such as THz lasers, THz photodetectors, and THz polarizers [28,29]. Additionally, antenna performance can also be improved by utilizing noval nanomaterials [30,31,32,33].

### 2.3. System Architecture

**Figure 5:**
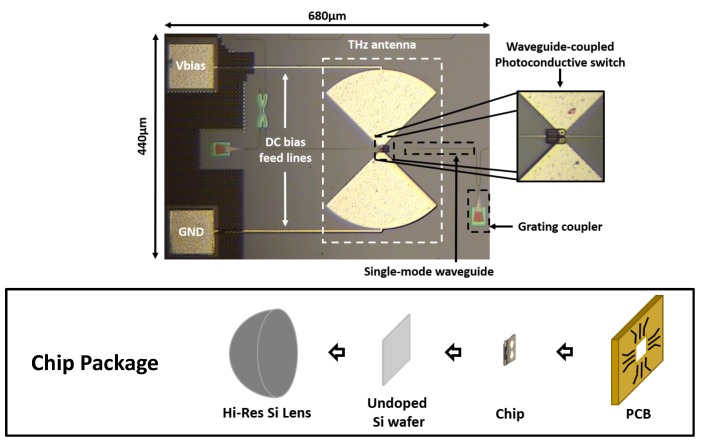
Micrograph of the prototype THz impulse radiator chip and chip package.

Figure 5 demonstrates the chip micrograph of the proposed Ge-based optical waveguide coupled THz impulse radiator using an SOI-based silicon photonics process technology. It occupies a small die area of 440 μm × 680 μm. A bowtie antenna is designed on the top metal layer to reduce conductive loss. Metal vias connect the photoconductive switch and the antenna. An integrated optical grating coupler with a typical insertion loss of 4.37 dB [26] is used to couple the free-space femtosecond laser into the on-chip waveguide, where the femtosecond laser propagates and is eventually absorbed by the photoconductive switch. Given that the length of the integrated waveguide is smaller than 300 μm in the prototype chip, its loss and dispersion effects are negligible, i.e., 0.05 dB loss and 1.3 fs pulse-width broadening. The main focus of this work is to design the Ge-based waveguide-coupled photoconductive switch. Therefore, pre-designed optical grating couplers and optical waveguides in the process development kit (PDK) are used in the design phase. These passive components can be further improved through custom design (loss and dispersion effects are challenging in the large-scale system integration, where much longer waveguide routing is required. The loss and dispersion performance of integrated grating coupler and waveguide can be further improved by custom design, which, however, is not the focus of this paper.). The chip package is also shown in Figure 5. A highly resistive silicon lens is attached to the backside of the chip to increase radiation efficiency.

## 3. Measurement Results

**Figure 6:**
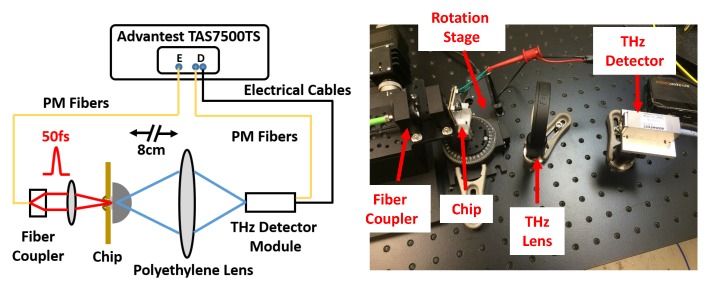
Measurement setup for the prototype THz impulse radiator chip.

The prototype THz impulse radiator chip was characterized in both time domain and frequency domain using an Advantest THz-TDS system (TAS7500TS), which is based on asynchronous optical sampling mechanism [34,35]. The characterization setup is demonstrated in Figure 6. A 50 fs pump laser beam from the Advantest THz-TDS system was coupled to the free space, and then focused onto the integrated grating coupler in the prototype chip. The focusing lens has a NA of 0.5. The chip was mounted on a rotation stage, which can be adjusted to achieve maximum coupling efficiency for the integrated grating coupler. A THz polyethylene lens focused the THz radiation to the THz detector. To measure average radiated power of the prototype chip, a calibrated pyroelectric detector, which is sensitive from 20 GHz to 1.5 THz, was utilized with a mechanical chopper modulating the pump femtosecond laser beam in the free space.

**Figure 7:**
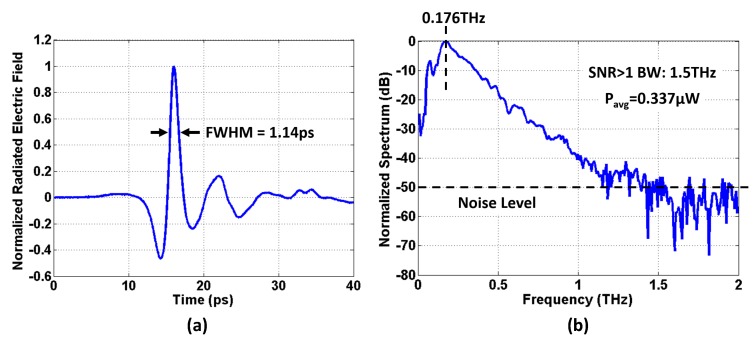
(**a**) Measured time-domain waveform of the radiated THz impulse. (**b**) Measured frequency-domain spectrum of the radiated THz impulse.

Figure 7 presents the measured THz impulse radiated by the prototype chip. With a maximum bias voltage of 3.5 V, the prototype chip radiates THz impulses with an FWHM of 1.14 ps. Its frequency spectrum, shown in Figure 7b, is obtained by performing DFT on the measured time-domain waveform. The radiated THz impulse has a peak frequency component at 176 GHz, and it has an SNR > 1 bandwidth of 1.5 THz. The measured average radiated power is 0.337 μW, and its DC-to-RF conversion efficiency is $3.6\times 10^{-5}$.

**Figure 8:**
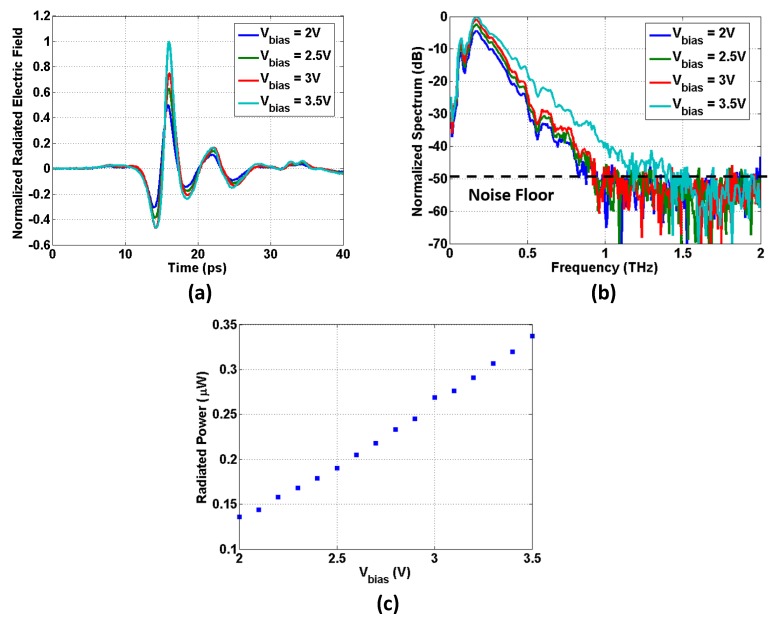
Measured effects of bias voltage on the radiated THz impulse. (**a**) Effects on the peak amplitude of the radiated THz impulse. (**b**) Effects on the SNR > 1 bandwidth of the radiated THz impulse. (**c**) Effects on the average radiated power.

Figure 8 demonstrates the measured effects of bias voltage on the radiated THz impulses. Theoretically, by increasing the bias voltage, the generated photocarriers in the Ge thin film have a higher drift velocity (before saturation happens), inducing a stronger transient current, and consequently, producing stronger THz impulse radiation. Measured results confirmed this theoretical prediction. When the bias voltage increased from 2 V to 3.5 V, the measured THz impulse had a larger peak amplitude (Figure 8a), a greater SNR > 1 bandwidth (Figure 8b), and a stronger average radiated power (Figure 8c) (we did not detect THz emission based on surface optical rectification [36,37] when the dc bias voltage is 0 V. One possible reason is that the waveguide-coupling mechanism, which forms a horizontal coupling interaction (with respect to the semiconductor substrate surface) between the optical excitation and the photoconductive material, may weaken the nonlinear optical rectification induced THz emission. Further investigation is needed). In this design, the maximum bias voltage is 3.5 V, and is limited by the current capacity of the electrical vias in the prototype chip.

## 4. Conclusions

In this work, an integrated Ge-based THz impulse radiator with an optical waveguide coupling scheme was implemented using an SOI-based silicon photonics process technology provided by IME A*STAR, Singapore. In the prototype chip, a phosphorus-doped Ge thin film is designed to reduce the photocarrier’s lifetime for faster transient response speed. Additionally, it enables the THz impulse radiator to operate with 1550 nm femtosecond lasers. The proposed optical waveguide coupling photoconductive switch provides a monolithic on-chip excitation scheme between the incident femtosecond laser and the photoconductive material. This monolithic configuration facilitates full-system integration of THz impulse radiators and other silicon photonics modules. The prototype CMOS-compatible THz impulse radiator chip can radiate 1.14 ps THz impulses with an SNR > 1 bandwidth of 1.5 THz and an average radiated power of 0.337 μW. The DC-to-RF conversion efficiency of the prototype chip is $3.6\times 10^{-5}$.

## References

[1] Siegel P.H. (2004). Terahertz technology in biology and medicine. IEEE Trans. Microw. Theory Tech..

[2] Siegel P.H. (2002). Terahertz technology. IEEE Trans. Microw. Theory Tech..

[3] Aggrawal H., Chen P., Assefzadeh M.M., Jamali B., Babakhani A. (2016). Gone in a Picosecond: Techniques for the Generation and Detection of Picosecond Pulses and Their Applications. IEEE Microw. Mag..

[4] Liu L., Jiang Z., Rahman S., Shams M.I.B., Jing B., Kannegulla A., Cheng L.J. (2016). Quasi-Optical Terahertz Microfluidic Devices for Chemical Sensing and Imaging. Micromachines.

[5] Alfihed S., Bergen M.H., Ciocoiu A., Holzman J.F., Foulds I.G. (2018). Characterization and Integration of Terahertz Technology within Microfluidic Platforms. Micromachines.

[6] Friederich F., von Spiegel W., Bauer M., Meng F., Thomson M.D., Boppel S., Lisauskas A., Hils B., Krozer V., Keil A. (2011). THz Active Imaging Systems With Real-Time Capabilities. IEEE Trans. Terahertz Sci. Technol..

[7] Chen P., Babakhani A. (2017). 3-D Radar Imaging Based on a Synthetic Array of 30-GHz Impulse Radiators With On-Chip Antennas in 130-nm SiGe BiCMOS. IEEE Trans. Microw. Theory Tech..

[8] Chen P., Babakhani A. A 30GHz impulse radiator with on-chip antennas for high-resolution 3D imaging. Proceedings of the 2015 IEEE Radio and Wireless Symposium (RWS).

[9] Malhotra I., Jha K.R., Singh G. (2018). Terahertz antenna technology for imaging applications: A technical review. Int. J. Microw. Wirel. Technol..

[10] Tonouchi M. (2007). Cutting-edge terahertz technology. Nat. Photonics.

[11] Nagatsuma T., Ducournau G., Renaud C.C. (2016). Advances in terahertz communications accelerated by photonics. Nat. Photonics.

[12] Chen P., Wang Y., Babakhani A. A 4ps amplitude reconfigurable impulse radiator with THz-TDS characterization method in 0.13 μm SiGe BiCMOS. Proceedings of the 2016 IEEE MTT-S International Microwave Symposium (IMS).

[13] Chen P., Assefzadeh M.M., Babakhani A. (2016). A Nonlinear Q-Switching Impedance Technique for Picosecond Pulse Radiation in Silicon. IEEE Trans. Microw. Theory Tech..

[14] Assefzadeh M.M., Babakhani A. Broadband THz spectroscopic imaging based on a fully-integrated 4 × 2 Digital-to-Impulse radiating array with a full-spectrum of 0.03–1.03THz in silicon. Proceedings of the 2016 IEEE Symposium on VLSI Technology.

[15] Assefzadeh M.M., Chen P., Babakhani A. High-power THz pulse radiation with GHz repetition rate in silicon. Proceedings of the 2016 41st International Conference on Infrared, Millimeter, and Terahertz Waves (IRMMW-THz).

[16] Assefzadeh M.M., Babakhani A. (2017). Broadband Oscillator-Free THz Pulse Generation and Radiation Based on Direct Digital-to-Impulse Architecture. IEEE J. Solid-State Circuits.

[17] Auston D.H. (1975). Picosecond optoelectronic switching and gating in silicon. Appl. Phys. Lett..

[18] Berry C.W., Jarrahi M. (2012). Terahertz generation using plasmonic photoconductive gratings. New J. Phys..

[19] Jooshesh A., Bahrami-Yekta V., Zhang J., Tiedje T., Darcie T.E., Gordon R. (2015). Plasmon-Enhanced below Bandgap Photoconductive Terahertz Generation and Detection. Nano Lett..

[20] Lepeshov S., Gorodetsky A., Krasnok A., Toropov N., Vartanyan T.A., Belov P., Alú A., Rafailov E.U. (2018). Boosting Terahertz Photoconductive Antenna Performance with Optimised Plasmonic Nanostructures. Sci. Rep..

[21] Yardimci N.T., Jarrahi M. (2018). Nanostructure-Enhanced Photoconductive Terahertz Emission and Detection. Small.

[22] Burford N.M., Evans M.J., El-Shenawee M.O. (2018). Plasmonic Nanodisk Thin-Film Terahertz Photoconductive Antenna. IEEE Trans. Terahertz Sci. Technol..

[23] Bashirpour M., Forouzmehr M., Hosseininejad S.E., Kolahdouz M., Neshat M. (2019). Improvement of Terahertz Photoconductive Antenna using Optical Antenna Array of ZnO Nanorods. Sci. Rep..

[24] Ding R., Baehr-Jones T., Pinguet T., Li J., Harris N.C., Streshinsky M., He L., Novack A., Lim A.E.J., Liow T.Y. A silicon platform for high-speed photonics systems. Proceedings of the OFC/NFOEC.

[25] Chen P., Hosseini M., Babakhani A. An integrated germanium-based optical waveguide coupled THz photoconductive antenna in silicon. Proceedings of the 2016 Conference on Lasers and Electro-Optics (CLEO).

[26] Novack A., Liu Y., Ding R., Gould M., Baehr-Jones T., Li Q., Yang Y., Ma Y., Zhang Y., Padmaraju K. A 30 GHz silicon photonic platform. Proceedings of the 10th International Conference on Group IV Photonics.

[27] Sekine N., Hirakawa K., Sogawa F., Arakawa Y., Usami N., Shiraki Y., Katoda T. (1996). Ultrashort lifetime photocarriers in Ge thin films. Appl. Phys. Lett..

[28] Ferguson B., Zhang X.C. (2002). Materials for terahertz science and technology. Nat. Mater..

[29] Komatsu N., Gao W., Chen P., Guo C., Babakhani A., Kono J. (2017). Modulation-Doped Multiple Quantum Wells of Aligned Single-Wall Carbon Nanotubes. Adv. Funct. Mater..

[30] Burke P.J. Carbon Nanotube Devices for GHz to THz Applications. Proceedings of the Optics East.

[31] Bengio E.A., Senic D., Taylor L.W., Tsentalovich D.E., Chen P., Holloway C.L., Babakhani A., Long C.J., Novotny D.R., Booth J.C. (2017). High efficiency carbon nanotube thread antennas. Appl. Phys. Lett..

[32] Bandaru P.R. (2007). Electrical Properties and Applications of Carbon Nanotube Structures. J. Nanosci. Nanotechnol..

[33] Bengio E.A., Senic D., Taylor L.W., Headrick R.J., King M., Chen P., Little C.A., Ladbury J., Long C.J., Holloway C.L. (2019). Carbon Nanotube Thin Film Patch Antennas for Wireless Communications. Appl. Phys. Lett..

[34] Yasui T., Saneyoshi E., Araki T. (2005). Asynchronous optical sampling terahertz time-domain spectroscopy for ultrahigh spectral resolution and rapid data acquisition. Appl. Phys. Lett..

[35] Chen P., Assefzadeh M.M., Babakhani A. (2017). Time-Domain Characterization of Silicon-Based Integrated Picosecond Impulse Radiators. IEEE Trans. Terahertz Sci. Technol..

[36] Chuang S.L., Schmitt-Rink S., Greene B.I., Saeta P.N., Levi A.F.J. (1992). Optical rectification at semiconductor surfaces. Phys. Rev. Lett..

[37] Peters L., Tunesi J., Pasquazi A., Peccianti M. (2018). High-energy terahertz surface optical rectification. Nano Energy.

